# Construct validation of judgement-based assessments of medical trainees’ competency in the workplace using a “Kanesian” approach to validation

**DOI:** 10.1186/s12909-015-0520-1

**Published:** 2015-12-30

**Authors:** D. A. McGill, C. P. M. van der Vleuten, M. J. Clarke

**Affiliations:** Department of Cardiology, The Canberra Hospital, Garran, ACT 2605 Australia; Department of Educational Research and Development, Maastricht University, Maastricht, The Netherlands; Clinical Trial Service Unit, University of Oxford, Oxford, UK

**Keywords:** Internal validity, Psychometrics, Workplace-based assessment, Medical education, Competency constructs, Clinical competence

## Abstract

**Background:**

Evaluations of clinical assessments that use judgement-based methods have frequently shown them to have sub-optimal reliability and internal validity evidence for their interpretation and intended use. The aim of this study was to enhance that validity evidence by an evaluation of the internal validity and reliability of competency constructs from supervisors’ end-of-term summative assessments for prevocational medical trainees.

**Methods:**

The populations were medical trainees preparing for full registration as a medical practitioner (74) and supervisors who undertook ≥2 end-of-term summative assessments (*n* = 349) from a single institution. Confirmatory Factor Analysis was used to evaluate assessment internal construct validity. The hypothesised competency construct model to be tested, identified by exploratory factor analysis, had a theoretical basis established in workplace-psychology literature. Comparisons were made with competing models of potential competency constructs including the competency construct model of the original assessment. The optimal model for the competency constructs was identified using model fit and measurement invariance analysis. Construct homogeneity was assessed by Cronbach’s α. Reliability measures were variance components of individual competency items and the identified competency constructs, and the number of assessments needed to achieve adequate reliability of *R* > 0.80.

**Results:**

The hypothesised competency constructs of “general professional job performance”, “clinical skills” and “professional abilities” provides a good model-fit to the data, and a better fit than all alternative models. Model fit indices were *χ*2/df = 2.8; RMSEA = 0.073 (CI 0.057-0.088); CFI = 0.93; TLI = 0.95; SRMR = 0.039; WRMR = 0.93; AIC = 3879; and BIC = 4018). The optimal model had adequate measurement invariance with nested analysis of important population subgroups supporting the presence of full metric invariance. Reliability estimates for the competency construct “general professional job performance” indicated a resource efficient and reliable assessment for such a construct (6 assessments for an *R* > 0.80). Item homogeneity was good (Cronbach’s alpha = 0.899). Other competency constructs are resource intensive requiring ≥11 assessments for a reliable assessment score.

**Conclusion:**

Internal validity and reliability of clinical competence assessments using judgement-based methods are acceptable when actual competency constructs used by assessors are adequately identified. Validation for interpretation and use of supervisors’ assessment in local training schemes is feasible using standard methods for gathering validity evidence.

## Background

The evaluations of judgement-based clinical performance assessments have consistently shown problems with reliability and validity [1, 2]. Documentation of the varying influences of context on assessment ratings [3], including the effect of rater experience [4], the type of assessor [5] and variability in understanding about the meaning and interpretation of competency domain constructs [6], highlight some of the issues about these important types of assessments. The validation of workplace-based assessments (WBAs) remains an area of ongoing improvement as identified by Kogan and colleagues: “Although many tools are available for the direct observation of clinical skills, validity evidence and description of educational outcomes are scarce” [2].

An argument-based approach to validation followed by evaluation, an approach long championed by Michael Kane [7–9], provides a framework for the evaluation of claims of competency based on assessment scores obtained from many different forms of assessment [10]. Within this framework, the educator states explicitly and in detail the proposed interpretation and use of the assessment scores, and these are then followed by evaluation of the plausibility of the proposals [10]. Such a framework is also supported by R L Brennan who argues validation simply equates to using interpretative/use arguments (IUAs) plus evaluations: “What is required is clear specifications of IUAs and careful evaluation of them” [11]. If claims of interpretation and use from an assessment cannot be validated, then “they count against the test developer or user” [11]. This theory framework for validation is potentially useful for the evaluation of new but also established methods of the assessment of postgraduate medical trainees. It should be noted that this approach is one of a number of validity theory proposals that continue to evolve [12–15].

Previously we have identified concerns about the validity of a former supervisor-based end-of-term assessment for pre-vocational trainees in one institution in Australia [16, 17]. A face-value claim for these supervisor assessments is the eligibility of a trainee for full registration as a competent medical practitioner. The pre-existing domains meant to be assessed were *Clinical Competence*, *Communication Skills*, *Personal and Professional Abilities*, and *Overall-rating*. If a trainee received an assessment indicating competence in these domains, as identified by the supervisor in each term, then they were suitable for full and unconditional registration. A further face-value claim from the assessment relates to the original concept of formative assessment. The trainee is given the same assessment half-way through a term as a feedback and learning assessment. Thus the feedback “score” with associated advice is provided as an improvement process. The basic assessment format continues in Australia although the competency items and domains identified have changed. Our previous observations questioned these face-value assumptions and raised the possibility of an alternate dominate competency domain with acceptable reliability, namely a *general professional job performance* competency construct [16, 17].

Validation of judgement-based assessments ideally should proceed systematically and iteratively within a theory base. Using Kane’s validation framework [10], an IUA can be provided that adequately represents the intended interpretation and use of the assessment, and how it will be evaluated, including checking its inferences and assumptions. The assessment of a *general professional job performance* competency construct is a potential valuable construct that can be used in any broader assessment program, though as one of many competencies expected in a well-trained medical practitioner. The presence of a general factor in performance independent of halo and other common method biases has theoretical support from observations in organisational psychology literature [18].

**Fig. 1:**
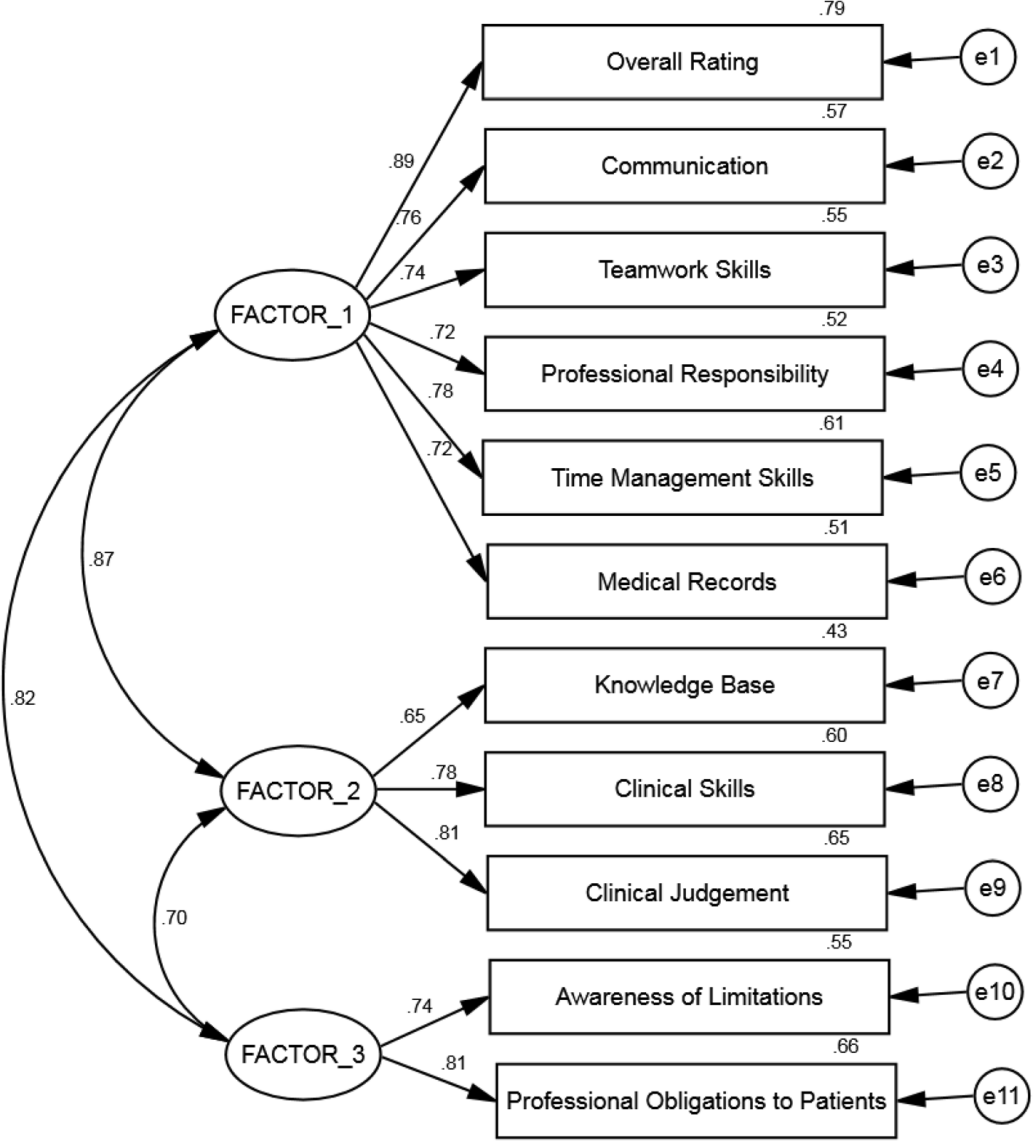
Optimal Model, Parameter Estimates and Error Estimates (Residual variances). (See Model Structure in the text for an explanation of the diagram)

Confirmatory factor analysis (CFA) is commonly used to evaluate internal construct validity of assessments. CFA is a structural equation modelling (SEM) method that uses directional hypothesis testing to evaluate the validity of non-directly observable (latent) constructs which are identified by observable variables or items. For example in Fig. 1, the competency domain *General Professional Job Performance* (Factor 1) is a latent competency concept that is hypothesised to be measurable by a number of observable behaviours and activities. CFA tests the directional hypothesis that an individual’s competency for this construct results in particular activities such a good medical record management, among other observable behaviours. That is, the presence of a high standard *General Professional Job Performance* competency results in the good medical record behaviour. If the directional relationship is confirmed in a CFA construct validation process, the measurable behaviours can then be used to confirm the presence and quality of a *General Professional Job Performance* competency for the trainee.

The aim of this study was to evaluate the internal validity and reliability of competency constructs for prevocational medical trainees, in particular to determine whether a potentially useful competency construct defined as a “general professional job performance” competency is valid and reliable for the particular context in which it was measured [17]. Individual training programs need to validate their own assessments, judgement-based assessments in particular, because such assessments relying on an individual’s judgement have no inherent transferrable reliability and validity. In Kane’s framework the assessment outcome measure needs to be valid for the context in which it is applied and what the result are used for [10].

## Methods

### Population and educational context

The population and context have previously been described [16, 17]. In brief, the populations are medical trainees preparing for unconditional registration and their supervisors who also undertake the assessment. Supervisors are specialty level consultants in a hospital network including secondary and tertiary level hospitals. The assessments used in this study were end-of-term and summative. Trainee scores for each assessment for each individual competency item are considered the primary unit of analysis. The assessment pro forma has been previously provided [16, 17]. A total of 74 trainees provided assessments with 64 trainees having 5, 12 had 4, and 2 had 3 assessments. Analysis was for supervisors with 2 or more assessments and only 6.3 % of all assessments involved only 1 supervisor leaving 349 usable assessments. Otherwise there were no exclusion criteria and all other assessments performed were included for all trainees, all supervisors and for all competency items assessed, as previously described [17].

Exploratory factor analysis, as a first-order model with correlated factors, provided the proposed constructs to be considered in the second-order factor model analysis using CFA [17]. The second-order model represents the hypothesis that the multiple seemingly distinct individual competency items, as described on an assessment form can be accounted for by one or more common underlying higher order constructs or domains. The individual competency items (observed variables) are the first-order variable and the factors (competency domains or constructs) are the second order variable in the model (Fig. 1).

### CFA

CFA is a form of structural equation modelling (SEM). SEM is used to test complex relationships between observed (measured) and unobserved (latent) variables and also relationships between two or more latent variables [19]. The purpose of the CFA is to examine a proposed measurement model and compare the model fit to other alternative models to ensure the proposed model is the most consistent with participants’ responses.

### Reliability

Each assessment competency item is the unit of analysis for each assessment (*n* = 349 assessment) and the reliability study has a single facet design with rater nested in trainee. The variance component for each observed competency item, the percent of variance for each trainee competency score and the individual item reliability coefficient (*R*-value) were estimated as previously described [16, 17]. Consistency of the item scores for the factors identified (competency domain constructs) was estimated by Cronbach’s alpha. The number of assessments to achieve a minimum acceptable reliability (NAAMAR) coefficient of ≥0.8 was calculated as a potential benchmarking statistic as previously described [16, 17].

### Sample size

An *a priori* evaluation indicated that the sample size is sufficient for a CFA analysis. Using an anticipated effect size of 0.1 as the minimum absolute anticipated effect size for the model; a statistical power level of 0.90; the number of latent variables of 3; the number of observed (indicator) variables of 11; and a probability level <0.05, then the minimum sample size for model structure is 129, and the minimum sample size to detect effect is 149 assessments.

### Missing data

Only 2.6 % of all scores (127 of 4886) contained missing values, an amount which normally would be considered low and be dealt with by simple methods such as trimming. However, the competency items Emergency Skills, Teaching and Learning and Procedural Skills accounted for 93 % (118/127) of all the missing values. Although Little's MCAR test [20] was non-significant (Chi-Square = 180.441, DF = 172, Sig. = .314) the pattern of distribution of the missing values indicated a non-random occurrence of missing values. Therefore these items were removed and analysis was with the remaining 11 competency items. Automatic imputation of missing score values was performed (IBM SPSS version 19). A repeat factor analysis using the subsequent values after imputation demonstrated the same factor structure and similar factor loadings.

### Assumptions

The assumption of non-normality was made for the CFA in view of the possibility of range restriction and other common method biases such as halo, leniency and stringency. The estimation method was the Mean- and Variance-adjusted Maximum Likelihood (MLMV).

### Model fit

Common fit indexes are Chi-square (*χ*^2^), the significance of *χ*^2^, the ratio of *χ*^2^ to degrees of freedom, Akaike information criterion (AIC), Bayes information criterion (BIC), Tucker–Lewis index (TLI), Comparative fit index (CFI), root mean square error of approximation (RMSEA with 95%CI), standardised root mean square residual (SRMR) and the weighted root mean residual (WRMR) [19, 21].

### Coefficients

The coefficients of hypothesized relationships and the significance of individual structural path relationships using z values associated with structural coefficients with the standard errors (SE) for standardised and unstandardized estimates are provided as an Mplus software Version 7.11 default.

### Sensitivity analysis by model comparisons

After examination of parameter estimates, fit indexes, and residuals, model comparisons and model modifications to the original hypothesized model were *a priori* planned to identify any possible better fitting and more parsimonious models [21].

### Measurement invariance

Evidence of whether construct validity is the same across 2 or more population groups will be evaluated by traditional methods to identify measurement invariance across groups [19, 22–24]. Demonstrating measurement invariance supports the use of the assessment across gender, race, and other demographically different subgroups that can be tested [25].

### Common method variance (CMV) analysis

CMV is common error variance shared among variables measured with and introduced as a function of the same method and/or source [26, 27]. The causes of CMV in rater-based assessments relate to issues such as leniency, stringency, range reduction of scores and halo effect. CMV was estimated using the correlation marker method and the unmeasured latent method construct (ULMC) approach. Since an *a priori* marker variable was not included in the original assessment, the variable with the smallest positive correlation in the data set was used as the maker [26] [27].

### Software

The original EFA was performed using IBM SPSS version 19 and the follow-on CFA was performed using Mplus Version 7.11 Muthen & Muthen. The path diagram was created with IBM AMOS version 21 which was also used as a sensitivity analysis for replicating the analysis and for measuring measurement invariance with an ML estimator.

### Ethics approval and consent

As only retrospective analyses of routinely collected and anonymised data were performed, the study was approved by ACT Health Human Research Ethics Committee’s Low Risk Sub-Committee approval number ETHLR.15.027. The ethics committee did not require consent to be obtained or a waiver of consent. The study was carried out in accordance with the Declaration of Helsinki. The anonymity of the participants was guaranteed.

## Results

### Descriptive statistics

Table 1 displays descriptive statistics and zero-order correlations for variables measuring trainee competence by their supervisor. Due to the large number of inter-correlations and the increased risk of a type I error, an adjusted a level of 0.001 was used to indicate significant bivariate relationships and model fit statistics. Correlations between items varied from 0.353 to 0.697, and all were significantly associated (*p* < 0.001).

**Table 1:**
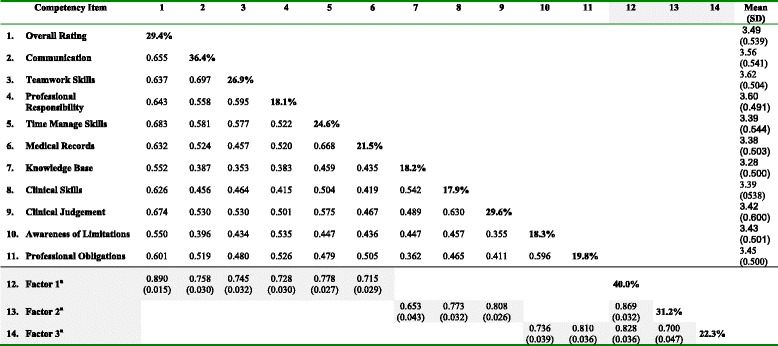
Descriptive statistics, correlations, and reliability results for the competency items, and the standardised estimates and reliability results of the modelled constructs

The diagonal cells contain percent variance for the score due to the trainee; all remaining variance is considered error variance; *p <* 0.001 for all correlations

All 2-tailed *p*-values <0.000; (see Fig. 1 for factor structure)

^a^Standardised Estimates of constructs with the items defining those constructs (SE) in shaded areas

### EFA Factor structure

The total variance accounted for increased to 71.9 % of total variance (full results available on request). Following imputation of the missing values the 3-Factor model accounted for approximately 73 % of the variance.

#### Measurement models

##### Confirmatory factor analysis

The hypothesised model tested was the factor structure identified after removal of potentially biasing competency items (Emergency Skills, Procedural Skills and Teaching and Learning), imputation of missing data, and the consolidation of Overall Rating, Time Management Skills, Medical Records, Communication skills, Teamwork Skills and Professional Responsibility attitude as the dominant first construct (Factor 1) called a “general professional job performance” competency construct. Factor 2 and Factor 3 were named “clinical skills” competency and “professional abilities” competency respectively. The standardised parameter-estimates with the standard error are presented in Table 1. All item loadings exceeded 0.60 and all differed reliably from zero (*p* < .0001).

##### Model structure

The hypothesised CFA model with continuous factor indicators is shown in the diagram (Fig. 1). The model has 3 correlated factors, with the first factor being measured by 6 continuous observed variables, the second measured by 3 and the third with 2 observed variables.

The ellipses represent the latent constructs (Factors). The rectangles are the observed variables (competency items). The circles are the error terms for each competency item. Bidirectional arrows between the factors indicate correlation with an assigned correlation coefficient (eg the correlation coefficient between factor 1 and factor 2 is 0.87). Unidirectional arrows indicate relationships that are predictive. For example, each of the first 6 observed variables are predicted by the latent variable (Factor 1), and the associated numbers are the standardised regression coefficients.

The directed arrows from the factors (latent variables) to the items (observed variables) indicate the loadings of the variable on the proposed latent factor. Each of the observed variables for the 3 latent competency domains has an associated error term (residual) which indicates that each observed variable is only partially predicted by the latent factor it is trying to measure. The rest is error. The numbers to the right of the observed variables are *R*-squared values (communalities in factor analysis), which is the proportion of variance explained by the latent competency factor for the individual item. An example of the interpretation of these numbers is that a one standard deviation increase on Factor 1 (job performance competence) is associated with a 0.89 standard deviation increase in the “overall rating” score, and is equivalent to a correlation of 0.89 between the factor and the observed variable. The amount of variance for the overall rating score explained by the competency construct (Factor 1) is 0.79 or 79 %. The same interpretation can be made for the results provided in Fig. 1 for all the individual item-Factor relationships.

##### Model fit

Parameter estimates obtained for the hypothesized measurement model are presented in Table 2, along with the model fit for other contending models available from the data and the context. The 3 Factor Model Factor structure from the EFA identifying a possible general job performance factor as described in Table 1 has the best model fit.

**Table 2:** Model Fit Indexes for alternative non-nested models

Model	Chi-squared (*χ*2)	Ratio of *χ*2 to df	Akaike information criterion (AIC)	Bayes information criterion (BIC)	Tucker–Lewis index (TLI)	Comparative fit index (CFI)	Root mean square error of approximation (RMSEA) (95%CI)	Standardised root mean square residual (SRMR)	Weighted root mean residual (WRMR)
Ideal Benchmark^a^	Non-significant *p*-value	<3; useful for nested models	Smaller the better; for model comparison (non-nested)	Smaller the better; for model comparison (non-nested)	≥ 0.95 id*e*al	≥ 0.95 ideal	<0.06 ideal;	≤ 0.08	< 0.90
					<0.90 reject	<0.90 reject	<0.08 acceptable; and with narrow 95 % confidence intervals		
3 Factor Model 1^b^	116.563 *p-*value <0.00	2.8	3879	4018	0.93	0.95	0.07	0.039	0.93
							(0.057–0.088)		
3 Factor Model 3^c^	223.258 *p-*value <0.00	3.0	4732	4906	0.89	0.91	0.08	0.048	1.14
							(0.067–0.090)		
3 Factor Model 4^d^	121.571 *p-*value <0.00	3.0	3884	4023	0.92	0.94	0.08 (0.060–0.091)	0.041	1.07
3 Factor Model^e^	211.42 *p-*value <0.00	2.85	4711	4884	0.90	0.92	0.07	0.045	1.06
							(0.062–0.085)		
1 Factor Model^f^	170.483 *p-*value <0.00	3.9	3955	4082	0.87	0.91	0.09 (0.077–0.105)	0.050	1.24
2 Factor Model^g^	139.489 *p-*value <0.00	3.2	3910	4041	0.91	0.93	0.08	0.043	1.11
							(0.066–0.095)		
1 Factor OC Model^h^	46.586 *p-*value <0.00	5.1	2103	2172	0.92	0.95	0.109	0.037	0.882
							(0.080–0.141)		

^a^From (Schreiber et al., 2006)

^b^3 Factor Model 1 = Factor structure from SPSS EFA identifying a possible general job performance factor as Factor 1

^c^3 Factor Model 3 = Factor structure from EFA using the *a priori* defined competency domains as 3 proposed Factors

^d^3 Factor Model 4 = Factor structure from SPSS EFA using the *a priori* defined competency domains as 3 proposed Factors but with potentially redundant items removed (Procedural, emergency and teach and learn)

^e^3 Factor model from original EFA with all 14 items

^f^1 Factor model with all 14 items

^g^2 Factor model with all 14 items

^h^1 Factor model with only those items within the “operational competence” construct and no other items

##### Model fit comparative analysis

As briefly stated in the introduction, the assessment was originally defined into 3 domains plus an “overall rating” item [17]. The original domains consisted of items thought to measure “clinical skills”, “communication skills”, and “professional competencies”. This original domain structure was analysed by CFA for a sensitivity analysis as a proposed explanatory structure, first with all the competencies and then again with the poorly performing items removed. Both model fit indices were less optimal than for the hypothesised model. When forced 1 and 2 factor models were evaluated, again the model fit indices were less optimal (Table 2). The parsimonious model with only 11 items and 3 factors, but with a factor 1 construct reflecting competencies consistent with general professional job performance had the best model fit.

##### Model parameters

The parameter indices for the optimal model reported in Table 1 are also illustrated by the standardized loadings (Fig. 1). The items’ loadings confirm that all of the 3 factors are well defined by the items. All the unstandardized variance components of the factors are statistically significant which indicates that the amount of variance accounted for by each factor is significantly different from zero. The *R*^*2*^ estimates which provide the amount of variance explained by the competency item are only moderate. The standardised variance explained by each item are all >0.50, except “knowledge”, indicating adequate although not ideal convergent validity. Also all residual correlations were low, ranging between 0 and 0.028, without any tendency to a positive and negative value (data not shown but available on request).

##### Reliability of the model

Sufficient internal consistency to use a composite of the scores as a measure of the different constructs was shown. Within a single level analysis, Cronbach’s alpha for Factor 1 was 0.899 (standardised alpha also 0.899), which indicates a high level of “internal consistency” for the scale with this specific sample within the context. Removal of any item results in a lower Cronbach's alpha. Cronbach’s alpha for Factor 2 was 0.786 (standardised 0.788) and for Factor 3 Cronbach’s alpha was 0.745 (standardised 0.745).

As an *a posteriori* evaluation a second-order factor analysis model was investigated with the first-order factors used as indicators of a second-order factor, that is, an overall latent variable at a higher level in a model structure with a third level. The model fit was not improved (Ratio of *χ*2 to df = 2.8; RMSEA = 0.073 (CI 0.057-0.088); CFI = 0.946; TLI = 0.927; SRMR = 0.039; WRMR = 0.93; AIC = 3879; and BIC = 4018).

The number of assessments needed to achieve an acceptable minimum reliability level of ≥ 0.80 remains essentially unchanged from previous observations [17] (Table 3). Only 6 assessments for construct 1 are needed to provide a reliable composite score for the construct expressed by the items.

**Table 3:** Reliability for Competency Items

Competency Item	Variance Components	Variances SEM^a^	Percent of Total Variance of trainees’ scores	Individual item Reliability Coefficient (*R*)	NAAMAR^b^
Overall Rating	0.084	0.016	29.4	0.676	10
Communication	0.104	0.017	36.4	0.741	7
Teamwork Skills	0.067	0.014	26.9	0.648	11
Professional Responsibility	0.043	0.013	18.1	0.557	19
Time Management Skills	0.071	0.018	24.6	0.620	13
Medical Records	0.054	0.015	21.5	0.578	15
Knowledge Base	0.045	0.015	18.2	0.527	17
Clinical Skills	0.051	0.014	17.9	0.522	18
Clinical Judgement	0.105	0.022	29.6	0.678	10
Awareness of Limitations	0.046	0.013	18.3	0.561	18
Professional Obligations	0.049	0.012	19.8	0.543	16
Competency Domain Construct 1	2.465		40.0	0.769	6
Competency Domain Construct 2	0.579		31.2	0.664	11
Competency Domain Construct 3	0.180		22.3	0.589	13

^a^ Standard Error of the Measurement

^b^NAAMAR = Number (rounded to digit) of assessments for adequate minimum acceptable reliability level of R = 0.80 with the NAAMAR calculated form the formula: *R* (reliability coefficient) = {σ^2^_subjects_ /(σ^2^_subjects +_ σ^2^_error_ /n)}, where n = assessments needed per trainee to attain the desired reliability coefficient

##### Measurement invariance

The model fit for all subgroups analysed as separate but nested groups was acceptable (Table 4). Testing for statistical invariance across nested sub-group comparisons (using AMOS and maximum likelihood estimator) indicated acceptable to moderately good model fit for all subgroups. This can be taken as support for configural invariance, i.e., equality in the number of latent factors across the major subgroups analysed. Testing for practical invariance across the subgroups also indicated acceptable comparisons with negligible difference in the CFI, TLI and SRMR between the respective groups, supporting the presence of full metric invariance (Table 4).

**Table 4:** Measurement invariance for nested model comparisons of major sub-groups^a^

Grouping	Model	df	χ^2b^	*χ* ^2^/df	RMSEA	CFI	TLI	SRMR	∆χ^2^	*p-*value for ∆χ^2^	∆CFI	∆TLI	∆SRMR
					(90 % CI)								
Female and Male Supervisors	Unconstrained	107	302.01	2.82	0.072	0.914	0.912	0.0746					
					(0.063–0.082)								
	All factor loadings constrained equal	118	323.37	2.74	0.071	0.910	0.916	0.0739	21.36	0.030	0.004	−0.004	0.0007
					(0.062–0.080)								
Female and Male Trainees	Unconstrained	107	296.97	2.775	0.072	0.916	0.914	0.0599					
					(0.062–0.081)								
	All factor loadings constrained equal	118	304.27	2.579	0.067	0.918	0.924	0.0601	7.299	0.774	0.002	−0.010	0.0002
					(0.058–0.077)								
Overseas (OTDs) and Australian Trained Doctors (ATDs)	Unconstrained	107	283.35	2.648	0.069	0.922	0.919	0.0718					
					(0.059–0.079)								
	All factor loadings constrained equal	118	301.60	2.556	0.067	0.918	0.924	0.0710	18.248	0.076	0.004	−0.004	0.0008
					(0.058–0.076)								

^a^Assuming models unconstrained to be correct

^b^All *p*-values <0.000 for the model *χ*2

*χ2* minimum fit function chi-square, *RMSEA* root mean square error of approximation, *CFI* comparative fit index, *TLI* Tucker-Lewis index, *SMSR* standardized root mean square residual, *Δ* parameter difference between constrained and unconstrained model

##### CMV analysis

The CMV analysis indicated that method bias was probably present. Partial Correlational marker method controlling for CMV using lowest item-item correlation (0.353) and the lowest item-factor (0.653) as the marker both demonstrated a reduction in the correlation although the correlations remained significant indicating that the relationships were still valid despite the CMV bias (results available on request). This was supported by the observations from the ULMC method with a reduction in all item-factor correlations after using a common factor ULMC analysis. Model-fit was also less optimal when adjusted for CMV (Ratio of *χ*2 to df = 4.6 with a change (Δ) = 1.3; AIC =2393; Δ *χ*2 = 49; TLI = 0.093; CFI = 0.095; RMSEA =0.095; and the SRMR -0.043). These observations indicate a probable confounding problem from CMV, but not enough to explain all the observed relationships.

## Discussion

This report provides further evidence that competency domain constructs identified by supervisors can be different to the competency domains presumed to have been assessed. The alternative constructs have internal validity and show measurement invariance between important subgroups of trainees. However, only one competency construct, defined as a “*general professional job performance”* competency, has a level of reliability that can be pragmatically applied, needing only 6 supervisor assessments to achieve an acceptable level of reliability. For the competency of “*general professional job performance*” trainees can be confident that their score interpretation is both precise and accurate if 6 assessments are obtained over a year.

A person competent in *general professional job performance* would be considered valuable in any very complex work context, especially when the health of other individuals is involved. In the workplace all the characteristics required for Factor 1 would be invaluable, namely: (1) *communication*: the “ability to communicate effectively and sensitively with patients and their families”; (2) *teamwork skills*: the “ability to work effectively in a multidiscipline team”; (3) *professional responsibility*: demonstrated through “punctuality, reliability and honesty”; (4) *time management skills*: ability to “organize and prioritize tasks to be undertaken”; (5) *medical records*: the ability to “maintain clear, comprehensive and accurate records”; and (6) linked to *overall rating*.

That these characteristics are identified by supervisors and are aggregated together as indicated in the correlative factor analysis, are identified as a theoretical possibility in the organisational literature, and confirmed in the internal validity analysis is not surprising. They are all characteristics of competency behaviours, when displayed by an individual could lead to positive effective outcomes within an organisational context, and be noticed by a supervisor. They would make work-life easier for the supervisor if applied optimally. These are also behavioural constructs that are not specific to medical practice or training, and would be expected to be identifiable in any complex professional workplace. They are also behavioural constructs that are commonly associated with professionalism in general [28].

Exploratory factor analysis has commonly been used as part of the evaluation of validity for global ratings of trainee competences in the past. Comparable evaluations from the past of supervisors who rated trainees’ competencies have made similar observations to those of this current study, as identified in our previous review [17]. Indeed, another more recent study of a similar Australian junior doctor population also found variation in the domain constructs of what was assessed compared to the domains expected to be assessed [29]. Moreover, from an Australian perspective, other evaluative research has identified concerns about the assessment of a similar junior doctor population in Australia [30–32], with observations indicating “that the tools and processes being used to monitor and assess junior doctor performance could be better” [32].

We have contributed to the literature, which we have reviewed previously [16, 17] by providing an evaluation of confounding influences on supervisor assessments, such as type of supervisor and gender for example, which has not been routinely undertaken in the validity evaluation of supervisor assessments. Similarly the use of CFA or other forms of SEM, with the addition of a reliability analysis have not routinely been used for the validity evaluation of these types of global assessment methods but is clearly feasible.

### Practical implications

An important practical implication is that fewer assessments are needed to achieve a reliable score for a truly valid competency construct. The need for fewer assessments is valuable for resource use from the time perspective of the institution, supervisors and trainees.

We have also shown that it is feasible to identify a new main construct that supervisors are using in assessing trainees’ competence, to demonstrate that a previously used assessment method lacks validity evidence, and to simultaneously show that it is feasible to do so within a single training program.

In addition we have shown that it is possible to strengthen validation methods in local training programs by applying traditional methodology to the evaluation of what constructs supervisors are using. By strengthening validation methods the possibility to benchmark between institutions is also strengthened. Moreover, the quality of training may be improved by developing other valid competency constructs that supervisors can assess, allowing for an increase in the sampling of a broader range of competencies.

Also fine-tuning the quality of supervisors’ assessments is potentially resource effective by improving the assessment built into daily work and identifying areas needing improvement. The types of methods used in this study have the potential to evaluate the validity of assessments occurring in the “authentic clinical environment and aligning what we measure with what we do” [33].

The need to “develop tools to meaningfully assess competencies” [34] continues to evolve, especially for competency assessment in the workplace [33]. Carraccio and Englander raise the issue of local relevance of any assessment program: “Studying the implementation of assessment tools in real-world settings—what works and what doesn’t for the faculty using the tool—thus becomes as critical as demonstrating its reliability and validity” [33].

### Limitations of the analysis and observations

#### Generalisability of the observations

As with all such internal structure analyses for locally obtained data, these observations may not be generalizable and the analysis would need to be replicated within each individual assessment program. The conclusions are limited to the particular sample, variables, and time frame represented by the data-set [35]. The results are subject to selection effects which include bias imposed by the individuals, types of measures, and occasions within the sampled groups and the time performed. Such potential biases pose problems for all WBAs.

The response to the generalisability issue for WBAs is that each assessment process should be validated in each individual training program, and the only thing that can be generalised is the methodology. The process of gathering validity evidence is cyclical and should be part of a continuing quality assurance process. Gathering validity evidence and reporting the evidence to standard-setting bodies is now routine for training and leaning programs in general education [36], and is becoming accepted practice in medical education even though the requirements differ [37, 38].

#### Common method biases

Common method biases leading to CMV exists when some of the differential covariance among items is due to the measurement method rather than the latent factors [19]. The CMV analysis indicated the probability of some confounding effect by inflating the associations between the competency domain constructs and the items. However, the confounding by CMV does not account for all the variance. Because one of the major causes of CMV arises from obtaining the measures from the same rater or source, one way of controlling for it is to collect the measures of these variables from different sources [26]. That is by many different assessors. The reliability analysis provides guidance on how many are potentially needed as a minimum. Reducing the influence of confounding thus can be potentially achieved by developing assessment programs which utilise multiple sources for evidence of competency [39]. If at all possible, intermediate and high-stake decisions should be “based on multiple data points after a meaningful aggregation of information” and being “supported by rigorous organisational procedures to ensure their dependability” [40].

#### Other potential confounding

The tendency to be lenient or severe in ratings is not consistent across jobs and accuracy of performance assessment is in part situation specific [41]. Variation in validity of assessments may vary within training programs, including that related to the timing of the assessment, trainee improvement, term culture, type of training and so on. However, this is the case for all WBAs and the need to identify potential confounders will always be a perennial issue. The methods to do so and be applicable to individual training programs are an ongoing improvement goal for medical education.

## Conclusions

The validity and reliability of clinical performance assessments using judgement-based methods are acceptable when the actual competency constructs used by assessors are identified using standard validation methods, in particular for a *general professional job performance competency* construct. The validation of these forms of assessment methods in local training schemes is feasible using accepted methods for gathering evidence of validity.

### Availability of supporting data

We are willing to share the data should anyone ask you for it, and are prepared to work with any interested researches on the re-analysis of the data particularly if for a systematic review using participant level data.

## Abbreviations

IUA: interpretative/use arguments
CFA: Confirmatory factor analysis
SEM: Structural equation modelling
EFA: Exploratory factor analysis
NAAMAR: Number of assessments to achieve a minimum acceptable reliability
AIC: Akaike information criterion
BIC: Bayes information criterion
TLI: Tucker–Lewis index
CFI: Comparative fit index
RMSEA: Root mean square error of approximation
CI: Confidence intervals
SRMR: Standardised root mean square residual
WRMR: Weighted root mean residual
SE: Standard error
CMV: Common method variance
ULMC: Unmeasured latent method construct
MLMV: Mean- and Variance-adjusted Maximum Likelihood
